# Supplementary material for: Saudi secondary school science textbooks’ ability to inculcate a PISA-Informed Scientific Identity
Source: PLoS One. 2025 Oct 6;20(10):e0325542. doi: 10.1371/journal.pone.0325542 (PMC12500081; doi:10.1371/journal.pone.0325542)
Supplement: S1 File — (DOCX) [file pone.0325542.s001.docx]

**Table No. (1) shows the frequency and percentages of indicators of the dimensions of the PISA test (scientific competencies - procedural knowledge - cognitive knowledge)**

| **field** | **the**  Skills | **Indicators** | **First intermediate grade** | | **Second intermediate grade** | | **Third intermediate grade** | | **the total** | |
| --- | --- | --- | --- | --- | --- | --- | --- | --- | --- | --- |
|  |  |  | **repetition** | **ratio** | **repetition** | **ratio** | **repetition** | **ratio** | **repetition** | **Forgetfulness** |
| **The content of science books requires the learner to:** | | | | | | | | | | |
| **Scientific competencies** | **1. Scientific explanation of phenomena** | **Providing an explanation of scientific phenomena.** | **41** | **2.8** | **47** | **3.21** | **46** | **3.14** | **134** | **9.75** |
|  |  | **Linking causes and effects associated with phenomena.** | **27** | **1.8** | **24** | **1.64** | **26** | **1.77** | **77** | **5.25** |
|  |  | **Applying knowledge and skills related to the phenomenon in life situations** | **27** | **1.8** | **30** | **2.05** | **30** | **2.05** | **87** | **5.93** |
|  |  | **Predicting potential changes in closely related phenomena.** | **29** | **19.78** | **20** | **1.36** | **28** | **1.91** | **77** | **5.25** |
|  |  | **Formulating possible explanatory hypotheses for the expected effects of the phenomenon.** | **4** | **0.61** | **3** | **0.21** | **2** | **0.14** | **9** | **0.61** |
|  |  | **the total** | 128 | **8.73** | 124 | **8:45** | 132 | **9** | **384** | **26.19** |
|  | **2. Evaluation and design of scientific investigation** | **Asking precise scientific questions related to the scientific position.** | **2** | **0.14** | **3** | **0.20** | **1** | **0.07** | **6** | **0.41** |
|  |  | **Propose appropriate alternatives to investigate answers to scientific questions.** | **9** | **0.61** | **10** | **0.68** | **11** | **0.84** | **30** | **2.05** |
|  |  | **Comparing appropriate alternatives to investigate answers to scientific questions.** | **6** | **0.41** | **10** | **0.68** | **11** | **0.48** | **27** | **1.8** |
|  |  | **Generalizing the most scientifically accurate answers to deal with precise scientific questions related to the scientific position.** | **6** | **0.41** | **7** | **0.74** | **11** | **0.84** | **24** | 1.64 |
|  |  | **the total** | **23** | **1.57** | **30** | **2.05** | **34** | **2.55** | **87** | **5.9** |
|  | **3.** **Interpret data and evidence scientifically.** | **Interpret scientific data with justification.** | **52** | **3.58** | **50** | **3.41** | **52** | **3.58** | **154** | **9.89** |
|  |  | **Draw conclusions that are closely related to the variables of the educational situation.** | **60** | **0.68** | **49** | **3.34** | **53** | **3.62** | **162** | **11:05** |
|  |  | **Distinguish between scientific arguments based on evidence and proof and theoretical assumptions based on perceptions.** | **1** | **0.07** | **1** | **0.07** | **3** | **0.20** | **4** | **0.27** |
|  |  | **Evaluating the validity of scientific conclusions.** | **12** | **0.82** | **12** | **0.82** | **14** | **0.95** | **38** | **2.59** |
|  |  | **the total** | **125** | **8.53** | **112** | **7.64** | **122** | **8.32** | **359** | **24.49** |
| **Total sum of scientific competency indicators** | | | 276 | **18.82** | **266** | **18,14** | **288** | **19.65** | **830** | **56.62** |
| **procedural knowledge** | | **Distinguish between types of variables (independent, dependent, and control)** | **8** | **0.55** | **4** | **0.27** | **8** | **0.55** | **20** | **1.36** |
|  |  | **Carrying out scientific experiments according to precise steps necessary to conduct experiments .** | **53** | **3.62** | **61** | **4.16** | **55** | **3.75** | **169** | **11.53** |
|  |  | **Accurate measurement using appropriate tools** | **20** | **1.36** | **27** | **1.84** | **32** | **2.18** | **79** | **5.39** |
|  |  | **Use valid methods to collect data and represent it using tables, graphs, and charts.** | **48** | **3.27** | **39** | **2.66** | **43** | **2.93** | **130** | **8.87** |
| **Total sum of procedural knowledge indicators** | | | **129** | **8.8** | **131** | **78.92** | **138** | **9.41** | **398** | **27.42** |
| **cognitive knowledge** | | **Distinguish between the main components of science (concepts - laws - theories - models).** | **0** |  | **0** |  | **0** |  | **0** | **0** |
|  |  | **Commitment to unbiased scientific logic.** | **5** | **0.34** | **14** | **0.95** | **19** | **1,3** | **38** | **2.59** |
|  |  | **Using induction and deduction strategies in scientific reasoning.** | **73** | **4.98** | **80** | **5.46** | **65** | **4.43** | **218** | **14.87** |
|  |  | **Formulating and verifying hypotheses** | **11** | **0.75** | **4** | **0.27** | **5** | **0.34** | **20** | **1.36** |
| **Total for cognitive knowledge indicators** | | | **84** | **5.73** | **84** | **5.73** | **70** | **4.77** | **238** | **16.23** |
| **Total** | | | **489** | **33.36** | **481** | **32.81** | **496** | **33.83** | **1466** | **100%** |

- It is clear from Table (1 ) that scientific competencies appeared in the science books for the intermediate stage with a frequency of (830) repetitions and a percentage of (56.62%), while procedural knowledge appeared with a frequency of (398) repetitions and a percentage of (27.15), followed by scientific knowledge, which appeared with a frequency of (238) and a percentage of (16.23). Thus, scientific competencies appear more frequently and frequently in the science books for the intermediate stage than the fields of procedural knowledge and cognitive knowledge. This disparity in the appearance of the three fields in intermediate science books may be due to the nature of the field itself and the practices it includes that each skill requires. The scientific competencies include practicing three general skills, which include a number of (13) indicators. These skills are supported by the content of science books through modules that are repeated in their content, such as the module “What have you read?” which is repeated in every lesson, as it requires the student to infer some information and provide an explanation for some scientific phenomena from the content he has read, and the module “The introductory experiment,” which begins each chapter in the book and requires the learner to practice critical thinking after conducting the experiment and practicing the skill of scientific investigation. The module “The experiment,” which appears in every lesson and is often related to the learner’s reality, requires him to practice the skill of analysis in arriving at the results resulting from conducting the experiment and practicing the skill of scientific investigation. This is also done through the module “Investigation from Real Life,” which comes at the end of each chapter of the book and requires the student to conduct an investigation into a question from real life and practice the skill of data analysis, which includes presenting scientific explanation and comparing the results of the student and his colleagues, then summarizing what he has reached. This is also done through the module “Practical Experiment.” These are the experiments found in the practical experiments booklet that directly target investigative activities and include almost all the scientific practices targeted by the field of scientific competencies. Therefore, the modules in the books that target scientific competencies may be more than the modules that target the field of procedural knowledge and cognitive knowledge due to the nature of the field of scientific competencies.
- The most frequently repeated indicator in the field of scientific competencies was the indicator of extracting results closely related to the variables of the educational situation, with a frequency of (162) repetitions, followed by the indicator of interpreting scientific data with justification , with a frequency of (154) repetitions within the skill. Interpretation of data and evidence scientifically , then the indicator of providing an explanation for scientific phenomena with a frequency of (134) repetitions, then the less frequently repeated indicators, which are: The link between the causes and results associated with phenomena , the application of knowledge and skills associated with the phenomenon in life situations , and the prediction of possible changes closely related to phenomena, and their frequency ranged between (77-87) repetitions. As for the indicators that appeared with weak repetition and need more focus on them in science books, they are: distinguishing between scientific arguments with evidence and proofs and theoretical assumptions based on perceptions, and it appeared with a frequency of (4) repetitions, and the indicator of posing precise scientific questions related to the scientific position , which appeared with a frequency of (6) repetitions, and the indicator of formulating possible explanatory hypotheses for the expected effects of the phenomenon, which appeared with a frequency of (9) repetitions, as well as the indicator of generalizing the most scientifically accurate answers to deal with precise scientific questions related to the scientific position, which appeared with a frequency of (24) repetitions, as well as the indicator of trade-offs between appropriate alternatives to investigate answers to scientific questions, which appeared with a frequency of (27) repetitions in the content of all science books in their three parts, which requires processing the content of these books to achieve these indicators among learners, and the indicator of posing appropriate alternatives to investigate answers to scientific questions, which appeared with a frequency of (30) repetitions, while the index of evaluating the validity of scientific conclusions appeared with a repetition of (38) repetitions.
- As for the field of procedural knowledge, which came in second place with a lower frequency than the field of scientific competencies, the reason for this may be that the indicators included in the field of scientific competencies are more than the indicators included in the field of procedural knowledge, which amounted to (4) indicators compared to (13) indicators in the field of scientific competencies. Also, the content of the books did not support the learner’s practice of distinguishing between the types of variables (independent, dependent, and controlling ) and did not target them sufficiently, as it is rarely required of him, despite the fact that the number of experiments included in the books amounted to (169) experiments, while the frequency of the appearance of this skill amounted to only (20) repetitions in the content of all the books of the stage in its three parts.
- The most frequently repeated indicator in the field of procedural knowledge was the indicator of implementing scientific experiments according to the precise steps necessary to conduct experiments, with a frequency of (169) repetitions, followed by the indicator of using correct methods to collect data and represent it using tables, graphs and charts , with a frequency of (130) repetitions, followed by the indicator of accurate measurement using appropriate tools, with a frequency of (79). These indicators were clearly targeted in the “ Introductory Experiment ” module , which begins each chapter in the book, and the “ Experiment ” module , which appears in each lesson, as well as through the “ Real-Life Investigation ” module , which comes at the end of each chapter of the book, as well as through the “ Practical Experiment ” module , which are the experiments found in the practical experiments booklet. All of these modules encourage the learner to implement scientific experiments according to precise steps that the learner adheres to when conducting experiments, and require him in several places to use accurate measurement using appropriate tools, whether that is by measuring temperatures or measuring materials and quantities used in conducting experiments, or by measuring areas and volumes, etc., and also often require the learner to make a graph or record numbers in Tables or data collection in a science notebook .
- As for the procedural knowledge component, which came in third place and with a lower frequency than the scientific competencies and procedural knowledge component, the reason may be due to the lack of targeting the practice of distinguishing between the main components of science (concepts - laws - theories - models), as this practice did not appear in the content of all science books for the stage. Also, targeting the practice of formulating and verifying hypotheses came with a very small frequency of (20) repetitions, which is an appearance that does not support the development of this practice well, as well as the practice of Commitment to unbiased scientific logic , which appeared repeatedly (38) in the content of all science books in their three parts, is also a repetition that does not adequately support the development of this practice among middle school students.
- The most frequently repeated indicator in the field of cognitive knowledge was the indicator of the use of induction and deduction strategies in scientific deduction. It appeared with good frequency, reaching ( 218 ) repetitions, through its direct targeting in the module “Unit Projects”, which comes at the beginning of each unit of the book, and requires the learner to search the Internet for a certain topic and present the information obtained through practicing the skill of induction and deduction, and through the module “What have you read?” through which the student practices induction and deduction strategies to present a scientific conclusion about the content that was read, as well as through the module “Science Notebook”, which begins each chapter of the book, as it requires the learner to describe certain information based on the processes of induction and deduction, as well as through the module “Experiment”, which appears in the lessons and requires the learner to analyze the educational situation and present conclusions based on practicing the skills of induction and deduction.
- As for the frequency of the fields’ appearance in the science books for the three grades, the frequency of their appearance was very close between the books of the three grades; as they appeared in order in the books of the third middle grade with a frequency of ( 496 ) repetitions and a percentage of ( 33.83 %), followed by the books of the first middle grade with a frequency of ( 489 ) repetitions and a percentage of ( 33.36 %), then followed by the books of the second middle grade with a frequency of ( 481 ) repetitions and a percentage of ( 32.81 %). By contemplating this result, it becomes clear to us that there is a great harmony between the content of the books with regard to the practices of scientific competencies, procedural knowledge and scientific knowledge.

Table No. (2) shows the frequencies and percentages of the indicators of the dimensions of the PISA test (content knowledge)

| **Content knowledge** | **Indicators** | **First intermediate grade** | | **Second intermediate grade** | | **Third grade middle school** | | **the total** | |
| --- | --- | --- | --- | --- | --- | --- | --- | --- | --- |
|  |  | **repetition** | **percentage** | **repetition** | **percentage** | **repetition** | **percentage** | **repetition** | **percentage** |
|  | **Physical and chemical systems ,** **It includes enabling the learner to know :** | | | | | | | | |
|  | **Structure and composition of matter - forms of chemical bonds and their properties.** | 8 | 0.85 | 10 | 1.01 | 36 | 3.83 | 54 | 5.75 |
|  | **Chemical reactions between substances - balanced chemical equations - examples of changes in matter The chemical .** | 9 | 0.96 | 1 | 0.10 | 27 | 2.88 | 37 | 3.94 |
|  | **Acids and bases and the differences between them - the change of substances due to acids and bases.** | **0** | **0** | **19** | **2.02** | **0** | **0** | **19** | **2.02** |
|  | **Physical properties of matter - Examples of physical changes - Materials that do and do not conduct heat and electricity.** | **21** | **2.24** | **22** | **2.34** | **20** | **2.13** | **63** | **6.7** |
|  | **The motion of matter and the forces acting on it - speed and friction - magnetic forces, gravity - electricity.** | **27** | **2.88** | **2** | **0.21** | **107** | **11.4** | **136** | **14.48** |
|  | **Energy and its transformations from one form to another - the law of conservation of energy .** | **0** | **0** | **35** | **3.73** | **12** | **1.23** | **47** | **5.01** |
|  | **Energy interactions with materials** | **7** | **0.75** | **2** | **0.21** | **9** | **0.96** | **18** | **1.92** |
|  | **Natural and artificial light sources - light, radio and sound waves .** | **2** | **0.21** | **17** | **1.81** | **4** | **0.43** | **23** | **2.45** |
|  | **the total** | **74** | **7.88** | **108** | **11.5** | **215** | **22.9** | **397** | **42.28** |
|  | **living systems ,**  **It includes enabling the learner to know:** | | | | | | | | |
|  | **Structure of living organisms - unicellular and multicellular organisms .** | **4** | **0.43** | **0** | **0** | **0** | **0** | **4** | **0.43** |
|  | **and activities of the cell - the difference between plant and animal cells .** | **18** | **1.92** | **2** | **0.21** | **26** | **2.77** | **46** | **4.9** |
|  | **Types of living organisms and their classification (plants - animals).** | **33** | **2.95** | **22** | **2.34** | **0** | **0** | **55** | **5.86** |
|  | **Components of the human digestive system and its components - enzymes.** | **0** | **0** | **15** | **1.6** | **0** | **0** | **15** | **1.6** |
|  | **Components of the respiratory and excretory system - functions - diseases.** | **0** | **0** | **26** | **2.77** | **0** | **0** | **26** | **2.77** |
|  | **Components of the skeletal system - Functions - Bone structure.** | **0** | **0** | **16** | **1.7** | **0** | **0** | **16** | **1.7** |
|  | **Components of the nervous system - how it works - its divisions - its safety.** | **0** | **0** | **21** | **2.24** | **0** | **0** | **21** | **2.24** |
|  | **Components of the circulatory and lymphatic systems - blood types and diseases - immunity and disease - infectious and sexually transmitted diseases.** | **0** | **0** | **36** | **3.83** | **0** | **0** | **36** | **3.83** |
|  | **Functions of the endocrine and reproductive system - Hormones - Male and female reproductive systems - Human development stages.** | **0** | **0** | **23** | **2.45** | **5** | **0.53** | **28** | **2.98** |
|  | **How does it work? The muscular system - human body movement - muscles and classification of muscle tissue** | **0** | **0** | **11** | **1.17** | **0** | **0** | **11** | **1.17** |
|  | **Types of diets and their importance** | **0** | **0** | **18** | **1.92** | **0** | **0** | **18** | **1.92** |
|  | **Structure and function in the cell and its activities - the difference between and biodiversity** | **0** | **0** | **0** | **0** | **46** | **4.9** | **46** | **4.9** |
|  | **Food chains and webs between creatures Living things in an ecosystem - the flow of energy through an ecosystem .** | **7** | **0.75** | **0** | **0** | **0** | **0** | **7** | **0.75** |
|  | **Components of the ecosystem and sustainability - renewable and non-renewable resources - and conservation projects** | **21** | **2.24** | **24** | **2.56** | **0** | **0** | **45** | **4.8** |
|  | **the total** | **83** | **8.84** | **214** | **22.79** | **77** | **8.2** | **374** | **39.83** |
|  | **Earth and space systems , including enabling the learner to know :** | | | | | | | | |
|  | **The structure of the Earth and its lithosphere**  **My watery water** | **61** | **6.5** | **0** | **0** | **0** | **0** | **61** | **6.5** |
|  | **Earth's atmosphere** | **12** | **1.28** | **0** | **0** | **0** | **0** | **12** | **1.28** |
|  | **History of Planet Earth - Evolution and Origins of Fossils - Historical Epochs of Earth.** | **4** | **0.43** | **0** | **0** | **0** | **0** | **4** | **0.43** |
|  | **Manifestations of hazards on Earth. - Earthquakes, volcanoes, plate movement .** | **13** | **1.38** | **0** | **0** | **59** | **6.28** | **72** | **7.68** |
|  | **Components of space - Solar system - Galaxies.** | **13** | **1.38** | **0** | **0** | **0** | **0** | **13** | **1.38** |
|  | **History of the universe and its cosmic measurements: - Calculating the distance between stars and galaxies - The concept of the light year .** | **6** | **0.64** | **0** | **0** | **0** | **0** | **6** | **0.64** |
|  |  | **109** | **11.61** | **0** | **0** | **59** | **6.28** | **168** | **17.89** |
|  | **Total** | **266** | **28,33** | **322** | **34.29** | **351** | **37.38** | **939** | **100%** |

- It is clear from Table ( ) that the fields of content knowledge appeared in the highest frequency in the field of physical and chemical systems with a frequency of (397) repetitions and a percentage of (42.28%), and the field of living systems appeared in second place with a frequency of (374) and a percentage of (39.82), and in last place came the field of earth and space systems with a frequency of (168) and a percentage of (17.89) .
- The most frequently repeated indicators in the field of physical and chemical systems were the index of the movement of matter and the forces affecting it - speed and friction - magnetic forces, gravity - electricity with a frequency of (136) and a percentage of (14.48), followed by the index of the physical properties of matter - examples of physical changes - materials that can and cannot conduct heat and electricity with a frequency of (63) repetitions and a percentage of (6.7), then the index of the structure and composition of matter - forms of chemical bonds and their properties with a frequency of (54) and a percentage of (5.75), then the rest of the indicators, the frequency of their appearance ranged between (18-37) repetitions and between percentages (1.92-3.94%), which means that all the indicators of the targeted cognitive content in the PISA test appeared in the content of science books for the intermediate stage, despite the difference in the frequency of their appearance.
- The most frequently repeated indicator in the field of living systems was the indicator of types of living organisms and their classification (plants - animals), with a frequency of occurrence of (55) and a percentage of (5.86%), followed by the indicator
