# Supplementary figures and images for: Saudi secondary school science textbooks’ ability to inculcate a PISA-Informed Scientific Identity
Source: PLoS One. 2025 Oct 6;20(10):e0325542. doi: 10.1371/journal.pone.0325542 (PMC12500081; doi:10.1371/journal.pone.0325542)

## Slide 1
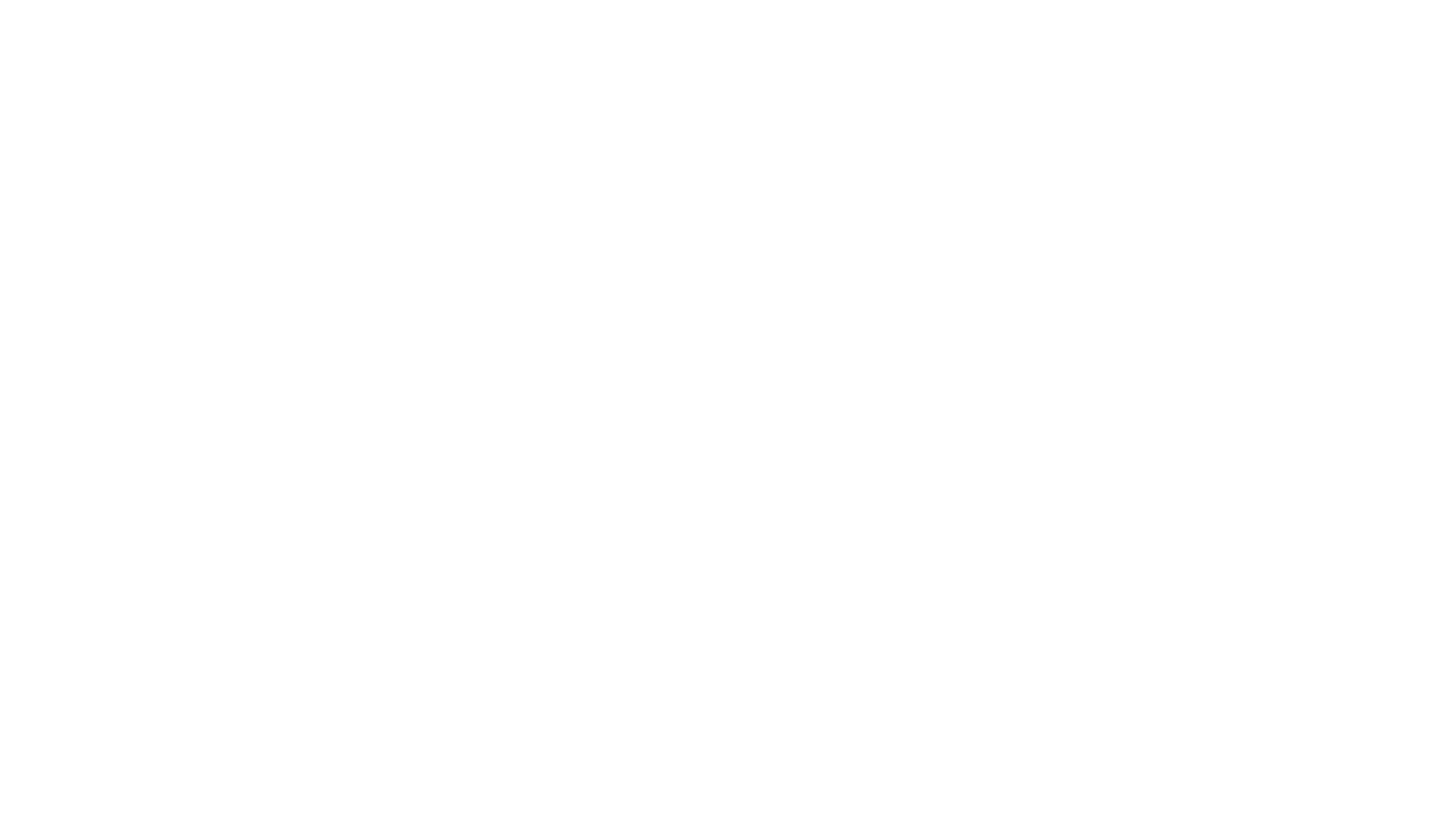

#

## Slide 2
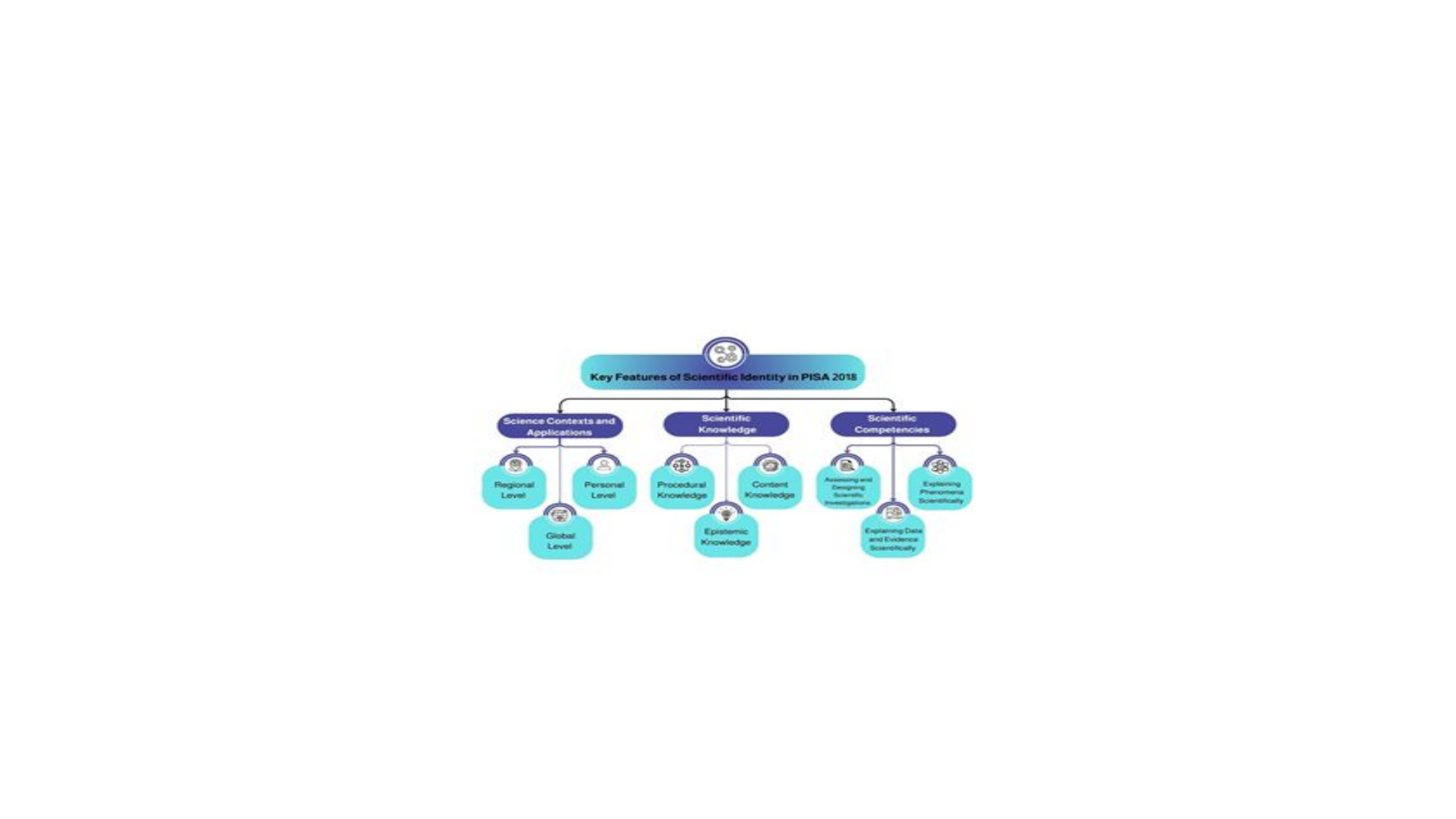

#

Supplement: S1 Fig — (PPTX) [file pone.0325542.s002.pptx]
