# Supplementary material for: Saudi secondary school science textbooks’ ability to inculcate a PISA-Informed Scientific Identity
Source: PLoS One. 2025 Oct 6;20(10):e0325542. doi: 10.1371/journal.pone.0325542 (PMC12500081; doi:10.1371/journal.pone.0325542)

PLOS ONE

Saudi Secondary School Science Textbooks' Ability to Inculcate a PISA-Informed Scientific Identity

--Manuscript Draft--

**Manuscript Number:**	PONE-D-25-08148
**Article Type:**	Research Article
**Full Title:**	Saudi Secondary School Science Textbooks' Ability to Inculcate a PISA-Informed Scientific Identity
**Short Title:**	School Science Textbooks'
**Corresponding Author:**	Amani Khalaf Hamdan Alghamdi<br>Imam Abdulrahman Bin Faisal University<br>Dammam, SAUDI ARABIA
**Keywords:**	Scientific identity; PISA, Saudi secondary science textbooks, content analysis
**Abstract:**	Saudi Arabia's low performance in the 2018 Programme for International Student Assessment (PISA) science tests (M =386 compared to M =489 overall) prompted a content analysis of Saudi secondary school science textbooks (N = 9, three books for each of Grade, 7, 8, and 9) (86% intracoder reliability). Using a definition of scientific identity formed using PISA's conceptual framework, we recommend textbook revisions that ensure more balanced coverage to offset the current weighing of scientific knowledge (55%), scientific competencies (28%), and science context (17%). Right now, students receive substantial coverage of major facts, concepts, and theories (knowledge) but less so competencies (proficiency at scientific explanations, design, evaluation, and interpretation), and how to understand and use science in different contexts at different levels (i.e., personal, local, and global). We assumed that students' appreciation that "science is for me" (i.e., positive scientific identity) will happen if they are evenly exposed to all PISA dimensions in secondary school. Because grade level profiles were very similar (what was present and missing), rebalancing must occur in all three grades. Revised science textbooks should positively influence Saudi secondary student's scientific identity and improve PISA performance.
**Order of Authors:**	Amani Khalaf Hamdan Alghamdi
	Wadha H. Al Otaibi
**Additional Information:**	
**Question**	**Response**
**Financial Disclosure**<br><br>Enter a financial disclosure statement that describes the sources of funding for the work included in this submission. Review the submission guidelines for detailed requirements. View published research articles from *PLOS ONE* for specific examples.<br><br>This statement is required for submission and **will appear in the published article** if the submission is accepted. Please make sure it is accurate.	Yes

Powered by Editorial Manager® and ProduXion Manager® from Aries Systems Corporation

**Funded studies**<br>Enter a statement with the following details:<br>• Initials of the authors who received each award<br>• Grant numbers awarded to each author<br>• The full name of each funder<br>• URL of each funder website<br>• Did the sponsors or funders play any role in the study design, data collection and analysis, decision to publish, or preparation of the manuscript?<br><br>Did you receive funding for this work?	
Please add funding details.<br>as follow-up to "**Financial Disclosure**<br><br>Enter a financial disclosure statement that describes the sources of funding for the work included in this submission. Review the submission guidelines for detailed requirements. View published research articles from *PLOS ONE* for specific examples.<br><br>This statement is required for submission and **will appear in the published article** if the submission is accepted. Please make sure it is accurate.<br><br>**Funded studies**<br>Enter a statement with the following details:<br>• Initials of the authors who received each award<br>• Grant numbers awarded to each author<br>• The full name of each funder<br>• URL of each funder website<br>• Did the sponsors or funders play any role in the study design, data collection and analysis, decision to publish, or preparation of the manuscript?<br><br>Did you receive funding for this work?"	Princess Nourah University<br>Deanship of Scientific Research
Please select the country of your main research funder (please select carefully as in some cases this is used in fee calculation).	SAUDI ARABIA - SA

as follow-up to "**Financial Disclosure**<br><br>Enter a financial disclosure statement that describes the sources of funding for the work included in this submission. Review the submission guidelines for detailed requirements. View published research articles from *PLOS ONE* for specific examples.<br><br>This statement is required for submission and **will appear in the published article** if the submission is accepted. Please make sure it is accurate.<br><br>**Funded studies**<br>Enter a statement with the following details:<br>• Initials of the authors who received each award<br>• Grant numbers awarded to each author<br>• The full name of each funder<br>• URL of each funder website<br>• Did the sponsors or funders play any role in the study design, data collection and analysis, decision to publish, or preparation of the manuscript?<br><br>Did you receive funding for this work?"	
**Competing Interests**<br><br>Use the instructions below to enter a competing interest statement for this submission. On behalf of all authors, disclose any competing interests that could be perceived to bias this work—acknowledging all financial support and any other relevant financial or non-financial competing interests.<br><br>This statement is required for submission and **will appear in the published article** if the submission is accepted. Please make sure it is accurate and that any funding sources listed in your Funding Information later in the submission form are also declared in your Financial Disclosure statement.	The authors have declared that no competing interests exist.

Powered by Editorial Manager® and ProduXion Manager® from Aries Systems Corporation

<table>
<tr><td>View published research articles from PLOS ONE for specific examples.

NO authors have competing interests

Enter: The authors have declared that no competing interests exist.

Authors with competing interests

Enter competing interest details beginning with this statement:

I have read the journal's policy and the authors of this manuscript have the following competing interests: [insert competing interests here]

* typeset</td><td></td></tr>
<tr><td>Ethics Statement

Enter an ethics statement for this submission. This statement is required if the study involved:

• Human participants
• Human specimens or tissue
• Vertebrate animals or cephalopods
• Vertebrate embryos or tissues
• Field research

Write "N/A" if the submission does not require an ethics statement.

General guidance is provided below. Consult the submission guidelines for detailed instructions. Make sure that all information entered here is included in the Methods section of the manuscript.</td><td>No human involved</td></tr>
</table>

**Format for specific study types**<br><br>**Human Subject Research (involving human participants and/or tissue)**<br>• Give the name of the institutional review board or ethics committee that approved the study<br>• Include the approval number and/or a statement indicating approval of this research<br>• Indicate the form of consent obtained (written/oral) or the reason that consent was not obtained (e.g. the data were analyzed anonymously)<br><br>**Animal Research (involving vertebrate animals, embryos or tissues)**<br>• Provide the name of the Institutional Animal Care and Use Committee (IACUC) or other relevant ethics board that reviewed the study protocol, and indicate whether they approved this research or granted a formal waiver of ethical approval<br>• Include an approval number if one was obtained<br>• If the study involved *non-human primates*, add *additional details* about animal welfare and steps taken to ameliorate suffering<br>• If anesthesia, euthanasia, or any kind of animal sacrifice is part of the study, include briefly which substances and/or methods were applied<br><br>**Field Research**<br><br>Include the following details if this study involves the collection of plant, animal, or other materials from a natural setting:<br>• Field permit number<br>• Name of the institution or relevant body that granted permission	
**Data Availability**<br><br>Authors are required to make all data underlying the findings described fully available, without restriction, and from the time of publication. PLOS allows rare exceptions to address legal and ethical concerns. See the PLOS Data Policy and FAQ for detailed information.	Yes - all data are fully available without restriction

A Data Availability Statement describing where the data can be found is required at submission. Your answers to this question constitute the Data Availability Statement and **will be published in the article**, if accepted.<br><br>**Important:** Stating 'data available on request from the author' is not sufficient. If your data are only available upon request, select 'No' for the first question and explain your exceptional situation in the text box.<br><br>Do the authors confirm that all data underlying the findings described in their manuscript are fully available without restriction?	
**Describe where the data may be found in full sentences. If you are copying our sample text, replace any instances of XXX with the appropriate details.**<br><br>• If the data are **held or will be held in a public repository**, include URLs, accession numbers or DOIs. If this information will only be available after acceptance, indicate this by ticking the box below. For example: *All XXX files are available from the XXX database (accession number(s) XXX, XXX.).*<br>• If the data are all contained **within the manuscript and/or Supporting Information files**, enter the following: *All relevant data are within the manuscript and its Supporting Information files.*<br>• If neither of these applies but you are able to provide **details of access elsewhere**, with or without limitations, please do so. For example:<br><br>*Data cannot be shared publicly because of [XXX]. Data are available from the XXX Institutional Data Access / Ethics Committee (contact via XXX) for researchers who meet the criteria for access to confidential data.*<br><br>*The data underlying the results presented in the study are available from (include the name of the third party*	None

and contact information or URL).<br>• This text is appropriate if the data are owned by a third party and authors do not have permission to share the data.<br><br>* typeset	
Additional data availability information:	Tick here if the URLs/accession numbers/DOIs will be available only after acceptance of the manuscript for publication so that we can ensure their inclusion before publication.

Powered by Editorial Manager® and ProduXion Manager® from Aries Systems Corporation

Manuscript

Click here to access/download;Manuscript;Scientific Identity Feb 25 with names.docx

Saudi Secondary School Science Textbooks' Ability to Inculcate a PISA-Informed Scientific Identity

Amani Khalaf. H. Alghamdi, Ph.D. SFHEA Corresponding Author

Professor of Curriculum and Pedagogy

Scientific Council, General Secretary

Department of Curriculum and instruction

College of Education

Imam Abdulrahman Bin Faisal University

Tel and Fax: (013) 333-37009

E-mail: akhalghamdi@iau.edu.sa

Alternative email: amani.k.hamdan@gmail.com

ID: A-3302-2015

Scopus ID: 22234006600

ORCID: http://orcid.org/0000-0002-8500-0266

Wadha H. Al Otaibi, Ph.D. First Author
Professor of Science Curriculum
Department of Curriculum and instruction
College of Education
Princess Nourah bint Abdulrahman University
wd.o@hotmail.com
whalotaibi@pnu.edu.sa

conflict of interest statement.

There is no conflict of interest

Abstract

Saudi Arabia’s low performance in the 2018 Programme for International Student Assessment (PISA) science tests (*M* =386 compared to *M* =489 overall) prompted a content analysis of Saudi secondary school science textbooks (*N* = 9, three books for each of Grade, 7, 8, and 9) (86% intracoder reliability). Using a definition of *scientific identity* formed using PISA’s conceptual framework, we recommend textbook revisions that ensure more balanced coverage to offset the current weighing of scientific knowledge (55%), scientific competencies (28%), and science context (17%). Right now, students receive substantial coverage of major facts, concepts, and theories (knowledge) but less so competencies (proficiency at scientific explanations, design, evaluation, and interpretation), and how to understand and use science in different contexts at different levels (i.e., personal, local, and global). We assumed that students’ appreciation that “science is for me” (i.e., positive scientific identity) will happen if they are evenly exposed to all PISA dimensions in secondary school. Because grade level profiles were very similar (what was present and missing), rebalancing must occur in all three grades. Revised science textbooks should positively influence Saudi secondary student’s scientific identity and improve PISA performance.

Keywords: Scientific identity; PISA, Saudi secondary science textbooks, content analysis

Introduction

The Organization for Economic Cooperation and Development (OECD) launched the Programme for International Student Assessment (PISA) in 2000. PISA aims to assess the ability of 15-year-old students (Grade 9) to use their knowledge and skills in reading, mathematics, and science to encounter life challenges. The test is administered once every three years based on unified criteria such as students' ages, regardless of their classes, applied questions, and impartiality of history and local, cultural factors. Due to COVID, PISA 2018 did not happen until 2022 (OECD, 2023). PISA has six performance (proficiency) levels ranging from low 1 to high 6. Higher levels 5 and 6 are more competent in abstract thinking, reasoning, argumentation, complexity, and dealing with uncertainty (OECD, 2023).

With its international framework, PISA is a valuable international study in the education field (Ababneh & Abdel Samad, 2018). Administered by OECD, it focuses on 15-year-old students' (Grade 9) learning, which is a significant indicator of their performance at age 19 when they traditionally enter the work force or enroll in higher education (Kastberg et al., 2021). PISA test results consistently show high and accurate confidence when measuring students' abilities (Mamedova et al., 2021; Polat et al., 2022). And PISA results are not affected by cultural factors or the surrounding environment of participating countries (Çikirikçi & Ulutas, 2015).

We are interested in the science aspect of the PISA because, as we will discuss, Saudi

... students' performance was unsatisfactory in 2018.

Arabian students presented with unsatisfactory results in 2018. Figure 1 summarizes the three main dimensions of PISA's science test in place for the 2018 test: scientific knowledge, scientific competencies, and scientific context (OECD, 2019, 2020). We draw on these to define and operationalize scientific identity. In brief, a scientific identity reflects the learner's ability and willingness to use their scientific knowledge and competency in practical situations in different contexts; they can identify with these three aspects of science and integrate them into their

persona. For us, science context is an important part of scientific identity because "science is used within a social and environmental context that involves consideration of economics, social behaviour, and [politics]" (OECD, 2023, p. 3).

Figure 1

Key Dimensions of Scientific Identity Informed by PISA's 2018 Science Test

OECD (2019) identified three contextual levels (i.e., personal, local/national, and global) and five specific contexts: health and disease; natural resources (e.g., conservation and responsible distribution); environmental quality (e.g., sustainability, pollution, and waste management); hazards and risks (e.g., lifestyle, changes to the earth and climate); and the frontiers (leading edge research) of science and technology. OECD (2019) chose these because (a) they are most relevant to Grade 9 students' lives and interests and (b) students' ability to bring their knowledge and competencies to these contexts enhances and sustains a nation's quality of life and relevant policy development. We worked up examples of scientific context and application for all three levels for use later in the study (see Table 1) (see also Al-Hoor, 2021; OECD, 2023; Polat et al., 2022; Somakim & Madang, 2016).

Table 1

Examples of the Scientific Context and Practical Application Aspect of Scientific Identity

**Scientific Application**	**Contexts**		
	**Personal**	**Regional (Local/National)**	**Global**
**Environmental Quality**	Protecting the environment through sustainably using raw materials and tools and going down the path that leads to environmental sustainment.	Knowing regional environments, available natural resources, and ways of protecting them.	Realising diverse biological variations, global environmental balance, and allies of dealing with global environmental problems.

**Health and Disease**	Adopting the required behavior for self-preservation, having good nutrition, and avoiding accidences and diseases. accidents?	Protecting health of society in line with the nature of diseases spread and having good health in society.	Dealing with epidemics spread worldwide and taking procedures to reduce their spread.
**Risks and Hazards**	Determining and avoiding risks that surround people at the personal level.	Dealing with risks that surround society (e.g., earthquakes, floods, and desertification.	Changes occur in the world because of risks of war, high temperatures, and climate change.
**Using Science and Technology**	Mastering technological skills in line with individuals' hobbies and skills	Using equipment and techniques of a positive effect in society.	Using related scientific equipment in discovering the universe, organisms, and diseases.
**Natural Resources**	Personal consumption of natural resources and energy.	Protecting natural resources in society, population structure, and appropriate resource distribution.	Achieving sustainable development and protecting global resources to achieve a global balance between renewable and non-renewable resources

Saudi Arabia's PISA Participation

Saudi Arabia's first PISA participation was in 2018, which is relatively late compared to other Arab nations. Jordan, Qatar, and Tunisia started in 2006. Saudi's 2018 PISA mean scores for reading, mathematics, and science were lower than overall performance for all nations (N = 79): Saudi reading M = 399 compared to M = 487; mathematics M =373 compared to M =487; and science M =386 compared to M =489 (Al-Harbi, 2020; Al-Zahrani, 2022). Most (38%) Saudi students achieved Level 2 or higher, which means they can provide scientific explanations in familiar contexts or draw conclusions based on simple investigations. However, none were proficient at Levels 5 or 6. These students can creatively and autonomously apply their science knowledge in a wide variety of situations, including unfamiliar ones (OECD, 2023).

Literature Review

Improving Saudi Arabia's science PISA performance will require work on all curricular products. In addition to curriculum, Depren (2018) and Gunaydin and Basaran (2022) found that

students’ reading comprehension, scientific content knowledge, attitude towards science, and the type of assessment and evaluation tool influenced their performance in PISA tests.

Regarding the science curriculum itself, Cansiz and Cansiz (2019) attributed Turkish students’ low PISA science performance to the nature of science curricula, which insufficiently reflected the scientific identity dimension of PISA tests (see Figure 1). Al-Towaisi (2020) confirmed that curricular content affected students’ PISA test performance followed *respectively* by educational policies, teacher performance, student self-competence, and schools’ surrounding environment. Al-Bashabesha (2016) and Mubarak (2010) similarly argued that students’ PISA science test performance was dependent on the nature and content of science curricula. Hepworth et al. (2013) argued that Irish students performed better on PISA than students from the other three United Kingdom countries because of Ireland’s science curricula excellence.

Studies have shown that well-developed instructional materials (including textbooks) can also improve students’ PISA performance in science and mathematics (Somakim & Madang, 2016) above and beyond the caliber of the entire curriculum. Given Egyptian students’ low PISA performance, Abdulfattah (2016) recommended developing science textbooks that take into account PISA frameworks. Turk et al. (2021) blamed Turkey’s low 2015 PISA performance on the textbooks. Students performed at PISA levels 1-4 but not the two higher levels. Turkish science textbook content focused on Bloom’s three lower levels of learning (remember, understand, and apply - not analyze, evaluate, and create). Sothayapetch et al. (2013) compared Finland and Thailand and found that the latter focused on the scientific method (process) while Finland focused on scientific concepts and contexts. Finland always scores higher than Thailand on PISA. Sothayapetch et al. recommended focusing on and analyzing science textbooks to discern scientific facts, concepts, context, theories, and contexts.

Al-Zahrani (2022) offered suggestions for improving Saudi Arabia's unsatisfactory PISA performance: redevelop curricula, augment teachers' performance, improve students' willingness to learn science, and prepare learning environments conducive to science learning. But given the research affirming the powerful role that textbook content plays on PISA performance, we were interested in how well existing Saudi secondary science textbooks can contribute to students gaining a scientific identity, so they can prepare for the future and perform better on PISA.

Scientific Identity

Saudi's less-than-satisfactory performance on the last PISA test, especially in the science realm, prompted our concern. It seems Saudi students are not identifying with science in their lives. We are convinced that gaining information about the content of Saudi textbooks relative to PISA's test parameters (see Figure 1) would serve as a primary reference point for a series of procedural steps to prepare Saudi students for future PISA tests and for their own future as Saudi and global citizens.

We were encouraged by several studies that reported that students' acquisition of a Science, Technology, Engineering, and Mathematics (STEM) identity had a positive effect on their desire to continue studying STEM topics, transferring learning into their professional life, and mastering scientific skills and research motivation (Al-Faqawi, 2002; Atkins et al., 2020; Deemer et al.,2022; Oseguera et al., 2019). We reasoned that a person with a *scientific identity* is also more inclined to participate in activities of a scientific community and appreciate the practical and functional value of science (Deemer, 2018).

Generally, a person's scientific identity effects their scientific activity, the development of professional goals containing science, and their future perceptions of science. Individuals acquire a scientific identity through iterative interactions with scientific contexts and social and functional situations. Once attained, they show positive attitudes toward scientific practices in a

way that reinforces a sense of belonging, pride, and high estimations of scientific knowledge and skills (Price et al., 2018; Shein et al., 2019; Young, 2018). As a caveat, we are aware that OECD (2020) recently expounded on how it conceptualizes a scientific identity, but this approach was not employed in our study, which drew instead on the PISA framework in place and intended for the 2018 testing event (see OECD, 2019).

We concur with OECD's (2020) position that "scientific identity is fundamental to science learning and attainment" (p. 12). We just defined and operationalized it differently in our study. We said scientific identity (i.e., whether people identify with science) depends on students' scientific knowledge, competencies, and context per OECD's (2019) framework. In contrast, OECD (2020) said scientific literacy is separate from scientific knowledge, competencies, and context. Scientific identity encompasses, instead, (a) the attitude that "science is for me" (science capital). (b) Students would have critical science agency – they would use science to address social inequalities. (c) They would view science as an ethics- and value-ladened practice. (d) And they would appreciate that science learning depends on inclusive and diverse representations and experiences of science (OECD, 2020).

Problem Statement, Research Question, and Study Objectives

Saudi Arabia's unsatisfactory performance on PISA tests in general, and science in particular, demands strategic efforts for improvement especially science curricula content and instructional materials like textbooks. Research shows that student's performance on PISA tests depends on the nature of national science curricula that they experienced before completing the test. Low performance on the PISA science test is indicative of an ill-developed scientific identity. This in turn suggests that inadequate textbook content pursuant to scientific knowledge, competencies, and context means a scientific identity alludes Saudi Grade 9 students. If so, there is a need to determine the extent to which existing textbooks contain content pursuant to a

This is vague. do you mean: This suggests that inadequate textbook content regarding scientific knowledge, competencies, and real-world contexts hinders the development of a scientific identity in Saudi Grade 9 students

scientific identity as defined in this study. To the best of our knowledge, research in this area is lacking in Saudi Arabia.

The main research question was thus, "*To what extent do Saudi secondary school science textbooks include scientific identity to prepare students for the future?*" Study objectives (i.e., tasks to collect data to answer the research question) included:

1. To determine the key dimensions of the PISA science test.

2. To conduct a content analysis of Saudi secondary level science textbooks issued in 2022–2023 to determine the presence of key PISA science dimensions

3. To judge the ability of Saudi secondary science textbooks to help students gain a scientific identity.

Study Significance

Using our empirical evidence of the extent to which building a scientific identity is present in Saudi secondary science textbooks, we intend to (a) propose a strategic vision around bolstering current levels of inclusion, (b) contribute to the strategic trend of developing Saudi science textbooks that entrench aspects of PISA's framework, (c) support Saudi education officials in efforts to improve the Kingdom's PISA performance and (d) affirm the scientific value of likeminded research for reading and mathematics. Developing Saudi curricular content so it aligns with PISA test content applies to science, reading, and mathematics (Al-Harbi, 2020; Al-Khadher, 2020; Al-Moqbel & Al-Omari, 2021; Hegras, 2010).

Conducting research about a nation's PISA performance is beneficial on several fronts. Research can generate information useful for curricular revisions and preparing students for future PISA tests. Said research can inform government-level development plans as well as changes to the education system. Research can inform efforts to create a school culture that supports students' PISA participation and, on a larger scale, socializes students to be active

citizens who use science to paint the state in a positive light. And attendant research results can help students gain a scientific identity they can take into the work force (Al-Towaisi, 2020; Depren, 2018; Gunaydin & Basaran, 2022; OECD, 2023).

Method

The descriptive-analytical approach we employed is well-suited for analyzing the content of science textbooks intended for Saudi Arabia's secondary school level. We strove to determine the extent to which the textbooks' content helped students gain a scientific identity as defined using PISA's (2019) three science dimensions: scientific knowledge, competencies, and context.

Document Sample Frame

write out numbers as words to express numbers up to nine (e.g., three, seven, eight).

nime

The sample frame comprised ~~*N* =~~ 9 approved science textbooks in use at the time of the study (three for each of Grades 7, 8, and 9). Although only Grade 9 students take the PISA test (OECD, 2023), we sampled all three levels as a preemptive strategy because, in addition to knowing what Grade 9 students were learning just before taking the PISA test, insights gained about the lower grades may prove useful in any future changes to Saudi secondary school science textbooks' content.

Study Procedures

To address the research question effectively, we adhered to the following structured steps: (a) identified PISA test dimension indicators, (b) developed and validated a content analysis tool and (c) applied the tool to conduct a content analysis of the document sample frame.

Identify PISA Scientific Identity Dimensions

As noted in the literature review (see Figure 1 and Table 1) we used PISA's (2019, see p. 103) three scientific dimensions to define scientific identity: (a) *scientific knowledge* (conceptual, procedural, and cognitive/epistemic); (b) *scientific competence* (explain scientific phenomena, evaluate and design scientific inquiry, and interpret scientific data and evidence); and (c) the

context of science and its practical applications (including health and disease, natural resource utilization, environmental considerations, potential risks, and the frontiers of science and technology).

Develop and Validate Content Analysis Tool

Using the information gained from step 1, we developed a content analysis coding tool that incorporated a comprehensive list of indicators associated with the PISA 2018 and PISA 2024 test dimensions (see OECD, 2019 [chapter 4], 2020). To validate the tool and ensure its accuracy, a group of science curriculum and teaching methods experts assessed the correlation of the indicators to the field it contained, integrity of linguistic formulation, and any modifications that would serve the study from their point of view. We heeded their observations, which included merging some indicators, separating others, deleting any duplicate or overlapping indicators, and modifying some language formulations. A satisfactory intracoder reliability coefficient (86%) was achieved with one researcher using the tool to analyze a science textbook sample twice within an interval of four weeks (Krippendorf, 1980).

Data Analysis

Content analysis entails counting things and placing them in categories (Krippendorf, 1980). The units of analysis within the documents included phrases, sentences, and paragraphs (including a series of paragraphs). As the content of each document was read and data were coded using the coding sheet (i.e., validated content analysis tool), data points were assigned to one of the three PISA dimensions: scientific knowledge, competencies, and context. Column and row frequencies and percentages were calculated for each document. Results are reported using descriptive statistics (frequencies and percentages).

Results and Discussion with Recommendations

The research question was "*To what extent do Saudi secondary school science textbooks include* s*cientific identity that prepares students for the future?"* In this study, we operationalized scientific identity as comprising PISA's scientific knowledge, competencies, and context (OECD, 2019). Table 2 profiles the overall content analysis results. The science textbooks contained mainly scientific knowledge (55%) followed by scientific competencies (28%) and science context and practical applications (17%). Discussion points and recommendations are integrated into presentation of results.

Table 2

Evidence of PISA's Three Dimensions in Saudi Secondary School Science Textbooks

**PISA Aspect of Scientific Identity:**	**Grade 7**		**Grade 8**		**Grade 9**		**Totals**	
	n=	%	*n=*	%	*n=*	%	*N=*	%
**Scientific Knowledge**	484	30	551	34	578	36	1613	**55%**
**Scientific Competencies**	276	33	266	32	288	35	830	**28%**
**Scientific Context**	122	25	211	43	154	32	487	**17%**
**Total**	882	**30%**	1028	**35%**	1020	**35%**	2930	100%

To answer the research question, the heavy incidence of one PISA dimension relative to the other two suggests that current textbooks lack the ability to ensure that Saudi secondary students gain a full scientific identity. They would be substantially exposed to major facts, concepts, and theories but less so competencies (proficiency at scientific explanations, design, evaluation, and interpretation), and how to understand and use science in different contexts at different levels. This imbalance might contribute to Saudi Grade 9 students saying, "Science is *not* for me." If this is the case, it could explain their unsatisfactory PISA science performance in

the 2018 test rounds (M =386 compared to the M =489 international average) (Al-Harbi, 2020; Al-Zahrani, 2022).

Scientific Knowledge

Scientific knowledge includes content, procedural, and epistemic knowledge. Content knowledge pertains to concepts, facts, theories, models, and ideas about the natural world that science has already established. Procedural knowledge concerns procedures that scientists use to apply the scientific method (empirical inquiry) to discover and establish the content knowledge. Epistemic or cognitive knowledge refers to what is involved in and what are the functions of questioning, observing, theorizing, hypothesizing, modelling, and argumentation. It also involves appreciating the different forms of scientific inquiry and the role impartial peer review plays in determining if content knowledge is true and can be trusted (OECD, 2019).

Per Figure 2, nearly two thirds (58%) of the scientific knowledge dimension in Saudi secondary science textbooks pertained to content knowledge followed by procedural knowledge (25%) and cognitive/epistemic knowledge (17%). This result affirms that all three forms of scientific *knowledge* were present in the textbooks, but representation was uneven. Al

Hawr (2021) found a similar pattern for Palestinian textbooks.

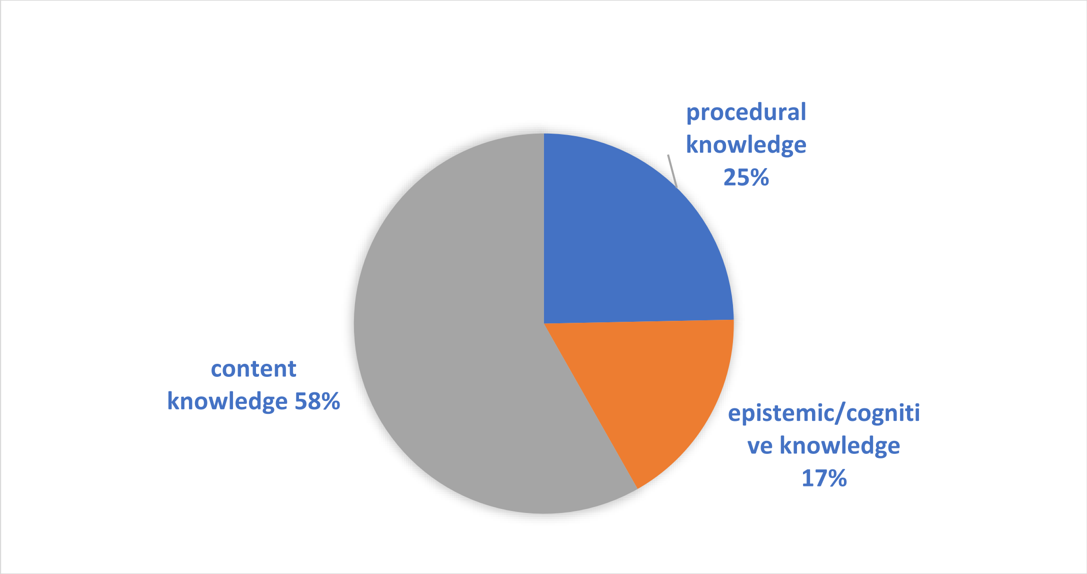

Figure 2

Percentage of PISA's Three Scientific Knowledge Components in Saudi Secondary Science Textbooks

Content knowledge in the science textbooks focused mostly on (a) *physical and chemical systems* (42%) (especially motion and force, and physical properties [50%]); (b) *living systems* (40%) (especially cell chemistry, genetics and biodiversity, conservation, and species classification [51%]); and (c) *earth and space systems* (18%) (especially plate movement, and earth's structure [79%]). Procedural knowledge mostly covered scientific experiments (42%) and data collection and representation (32%). Epistemic knowledge overwhelmingly (80%) dealt with drawing deductive and inductive scientific conclusions.

These results align with Al-Fahidi's (2021) study, which identified physical and chemical systems as the most recurrent areas in Saudi secondary science books followed by living systems, and then Earth and space systems. Similarly, Al Hawr (2021) (Palestinian study)

ranked physical and chemical systems at the top with variations in the rankings of living systems, and earth and space systems. Our results suggest, however, that when taught using the existing textbooks, Saudi secondary science students would miss out on certain aspects of scientific knowledge (low percentages 4–10%): research variables; main components of science (concepts, laws, theories, and models); formulating assumptions; acids and bases; energy interactions; light and sound; structure of organisms; food chains; history of the earth; and history of the universe. These components should be strengthened in future textbook revisions.

Scientific Competencies

The OECD (2019) clarified that its measurement of *scientific competency* reflects the scientific knowledge (i.e., content, procedural, and epistemic) "that 15-year-old students can reasonably be expected to have" (p. 101). Scientific competency comprises three factors: "the ability to explain phenomena scientifically, evaluate and design scientific enquiry, and interpret data and evidence scientifically" (p. 102). Respectively, students must be able to access and apply the scientific knowledge they hold to (a) recognize, offer, and evaluate scientific explanations for a range of natural and technological phenomena; (b) describe and appraise scientific investigations and inquiries (e.g., measurements, reliability, validity, and statistical confidence) and propose alternative ways to scientifically address questions; and (c) critically (using a skeptical disposition) analyze (look for patterns), evaluate (judge), and interpret (draw conclusions) scientific data, arguments, and claims from a variety of sources that employ a range of representations.

Scientific competencies in the Saudi secondary science textbooks focused equally on the ability to explain phenomena scientifically (46%) and interpret data and evidence scientifically (43%) and less so on evaluate and design scientific enquiry (11%) (see Figure 3).

This result affirms that all three forms of scientific competencies were present in the Saudi textbooks, but representation was skewed to two components (89%). Al Hawr (2021) reported similar results in Palestinian textbooks wherein the most recurrent scientific competencies were the ability to elucidate scientific phenomena followed very closely by the aptitude to clarify data and scientific evidence, and to a lesser extent the capability to assess and design scientific inquiry. Future Saudi textbook revisions should bolster the *evaluate and design scientific inquiry* component of scientific competencies. read this sentence again. This would highlight a critical and skeptical disposition, analyze to find patterns, make judgements, and draw scientific conclusions (OECD, 2019).

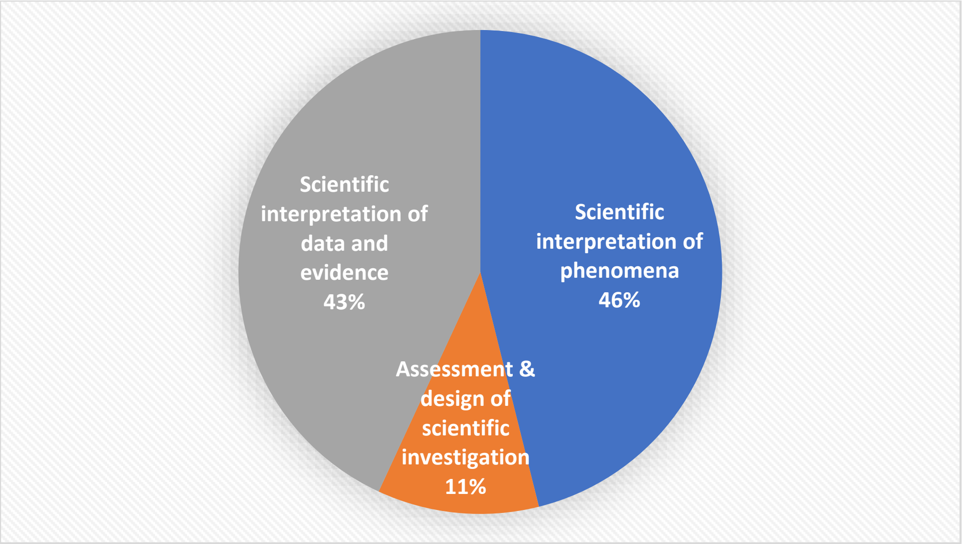

Figure 3

Percentage of PISA's Three Scientific Competency Components in Saudi Secondary Science Textbooks

To continue, the *scientific interpretation of phenomena* competency component focused mostly on providing an explanation of scientific phenomena (35%) followed by equal coverage (average 21% each) of link ing causes and consequences, apply ing knowledge in real life situations, and predict ing changes. The *scientific interpretation of data and evidence* component

provided fairly equal coverage of proposing alternative ways to address questions scientifically (34%), articulating tradeoffs between alternatives (31%), and generalizing answers to deal with specific questions (28%). The *assessment and design of scientific investigations* component focused equally on drawing conclusions (45%) and justifying data interpretation (43%).

We believe the organizational format of the Saudi textbooks contributed to the high presence on some PISA elements (OECD, 2019). Each book contained sections titled *unit projects, science notebook, the experiment, initiated experience, what did you read,* and *investigation from real life*. If Saudi secondary science students complete these learning tools, they, respectively, gain experience expounding on topics found on the internet, recording findings about phenomena in their science notebook, applying analytical skills to derive conclusions from their experiments, employing critical thinking to dissect and elucidate their results, deducing information and proffering scientific explanations for phenomena they have read about, and conducting real-life inquiries. Saudi secondary science teachers should continue to employ these practices on a regular basis and expand their usage to all aspects of the curriculum. Their use seemed to strengthen the possibility of developing a scientific identity.

That said, when taught using the existing textbooks, Saudi secondary science students would miss out on certain aspects of scientific competencies in each of the three components (low percentages 1–10%): distinguish between scientific arguments (1%), formulate hypotheses (2%), ask precise scientific questions (7%), and assess the validity of results (11%). These components should be strengthened in future textbook revisions. They require Bloom’s higher levels of cognitive learning (i.e., analysis, evaluation, and creation), which Turk et al. (2021) also found lacking in secondary science textbooks. This result may explain why Saudi students did not score at levels 5 and 6 in the 2018 PISA test (OECD, 2020).

Scientific Context

Our conceptualization of scientific identity included *scientific context* and its practical applications. OECD (2019) viewed scientific context as current and historical issues that demand some understanding of science and technology (see five contextual issues at Table 1). Also, “items in the PISA 2018 science assessment may relate to the self, family and peer groups (personal), to the community (local and national) or to life across the world (global)” (OECD, 2019, p. 103). Instead of assessing the context itself, the PISA test assesses students’ scientific knowledge and scientific competencies in specific contexts.

This means Saudi secondary science textbooks must include reference to these three levels and five contexts if students hope to pass this part of the PISA test and gain a richer sense of scientific identity as defined in our study. The content analysis revealed that although all three levels and all five contexts were evident in the textbooks, coverage focused mainly on two contexts: frontiers of science and technology (37%) and health and disease (30%) totaling more than two thirds (67%) (see Figure 4). The quality of the environment (15%), natural resources (11%), and risks and hazards (7%) received the least coverage.

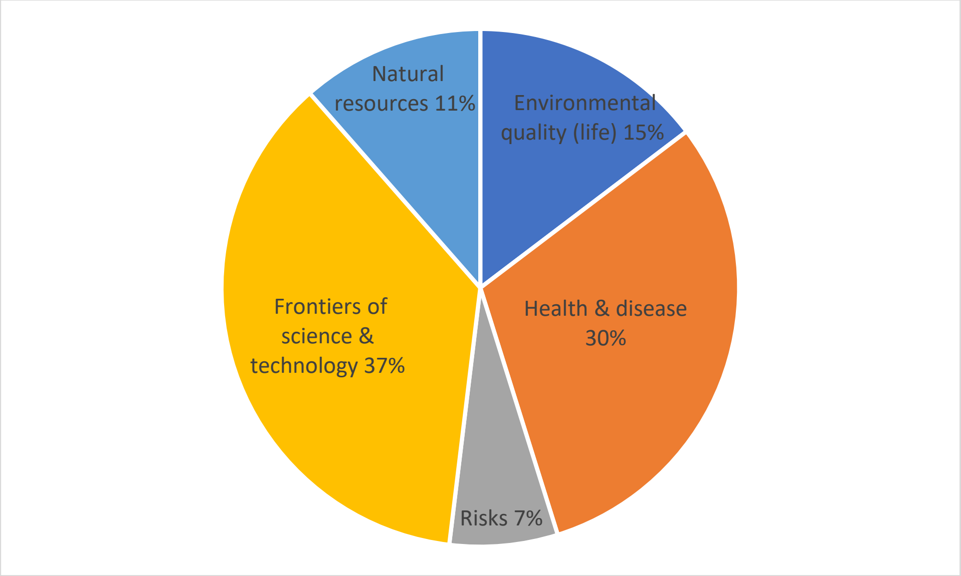

Figure 4

Percentage of PISA Scientific Contexts in Saudi Secondary Science Textbooks

We attribute the highest percentage for frontiers of science and technology (37%) to the textbooks' significant emphasis on developing students' research skills through the utilization of search engines and their ability to draw conclusions and acquire information across various fields. This emphasis is particularly evident in *Unit Projects*, which revolves around online research, and *Science via Websites*, which concludes each chapter and plays a crucial role in reinforcing these skills. The emphasis on health and disease (30%) likely reflects the textbooks' constant reference to students adhering to safety guidelines: maintaining cleanliness during experiments, safeguarding hands and eyes, and handling tools and materials with utmost care. These are reiterated in each chapter via sections on *initial experiment, experiment,* and *investigation from real life*. Indeed, most of the health and disease context scored for health (82%) and not disease (18%).

Three Contextual Levels

Regarding the three contextual levels, the majority (59%) of textbook content focused on the personal level (self, family, and peer groups), followed by global (24%) and local/national (community) (17%). This result suggests that the *scientific identity* of Saudi secondary science students is skewed to the personal level. They may not enter high school with a sense that they need scientific knowledge and competencies to deal with local/national (regional), and global issues with scientific overtones. They may not identify with the local, national, regional, and global from a scientific perspective. Taught using existing textbooks, Saudi science students cannot attain a scientific concern for the five contextual areas *beyond the personal*. Future revisions to Saudi secondary science textbooks must redress this issue so students' scientific identity extends to all three levels – personal, local/national, and global.

a noun is needed here; there are several adjectives and a noun is needed.

Grade Level Profiles

We collected data for all three secondary science grades even though only Grade 9 takes the PISA test. Results showed that the profile for each grade was very similar (what was present and missing) (see Table 2). Also, evidence of the textbooks' ability to inculcate a scientific identity, as conceptualized for our study, was present in all three grades at virtually the same level with Grade 7 nominally lower on account of less coverage of the scientific context (see Table 2). This result clearly suggests that any revisions to rebalance (accommodate what was missing) must occur in all three grades if inculcating a scientific identity is to succeed.

To that end, results suggest bolstering specific aspects of each separate PISA dimension. In scientific knowledge, add more information about procedural, and epistemic knowledge. In scientific competencies, add more on evaluate and design scientific enquiry. And

you had better change these imperative sentences and be less direct by using passive sentences.

in scientific context, add more on local and global levels and on more on disease, natural resources (e.g., conservation and responsible distribution); environmental quality (e.g., sustainability, pollution, and waste management); and hazards and risks (e.g., lifestyle, changes to the earth and climate). It is imperative that Saudi curricular designers and textbook authors reconsider PISA dimensions with limited recurrence and enhance their presence within future science textbooks.

Conclusion

The heavy incidence of one PISA dimension relative to the other two suggests that existing textbooks lack the ability to ensure that Saudi secondary students can gain a full scientific identity. We recommend that future Saudi Grade 7, 8, and 9 science textbooks should contain more balanced coverage of the three aspects of scientific identity to offset the current weighing of scientific knowledge (55%), scientific competencies (28%), and science context (17%). We reason that an appreciation of "science is for me" (i.e., a positive scientific identity) (OECD, 2020) will happen if learners are evenly exposed to all PISA dimensions. Others concur that improving science textbooks, so they align with PISA, is a recommended strategy for improving test performance (Abdulfattah, 2016; Sothayapetch et al., 2013; Turk et al., 2021) and, by association, scientific identity. With this new identity, students should be more inclined to partake in Saudi's scientific community and appreciate the value of science in society when they graduate (Deemer, 2018; Price et al., 2018; Young, 2018).

We further maintain that Saudi Arabia's PISA performance should improve over time because revised textbooks would be more aligned with test parameters (Al-Zahrani, 2022). Saudi scores for 2018 were in the 300 range (Al-Harbi, 2020; Al-Zahrani, 2022), but the normal distribution for PISA is 500 with a standard deviation of 100 points (OECD, 2019). Also, the

weakest textbook parts right now (especially epistemic knowledge) tend to require Bloom’s higher levels of learning, which are PISA’s higher proficiency levels (5 and 6). Saudi students did not achieve this proficiency on any science dimension (Al-Harbi, 2020; Al-Zahrani, 2022). Revised science textbooks should positively influence Saudi secondary student’s scientific identity and improve PISA performance.

References

Ababneh, G., & Abdel Samad, M. (2018). Gender gap in science achievement for Jordanian students in PISA 2015. *European Journal of Educational Research*, *7*(4), 963–972. https://doi.org/10.12973/eu-jer.7.4.963

Abdulfattah, S. (2016). tatwir tadris aleulum fi daw' maeayir PISA [Developing science teaching in light of PISA standards]. *Egyptian Journal for Scientific Education*, *19*(6), 29–64.

Abu Ouda, M. F., & Sumaya, F. (2022). mustawaa tadmin kutub aleulum lilmarhalat al'asasiat aleulya bi'abead PISA aldawliati [Level of inclusion in science textbooks of upper stage PISA dimensions]. *Jerusalem Open University Journal of Educational and Psychological Research and Studies*, *13*(38), 170–182.

Al-Bashabesha, K. (2016). astikshaf al'ada' altafadulii limutaghayir aljins fi PISA 2012 [*Exploring differential performance of the gender variable in PISA 2012*] (Master’s thesis, Mutah University, Jordan).

Al-Fahidi, H. (2021). taqyim muhtawaa kutub aleulum lilmarhalat almutawasitat fi almamlakat alearabiat alsaeudiat fi daw' almutatalabat almaerifiat libarnamaj PISA, 2018 [Evaluating the content of Saudi middle school science textbooks in light of PISA 2018 requirements]. *Journal of the Islamic University for Educational and Social Sciences*, 6, 1–53.

Al Hawr, D. (2021). maharat aikhtibar PISA fi kutub aleulum wamustawaa aiktisabiha waealaqatiha biaistikshaf almustaqbal ladaa talabat alsafi altaasie fi mudun aljanub alfilastinii (risalat majistir, jamieat al'aqsaa, Filastin [PISA test skills in science textbooks, level of acquisition and their relationship to southern Palestinian governorates' ninth-grade students' future outlook] [Unpublished master's thesis]. Al-Aqsa University, Palestine.

Al-Harbi, M. (2020). 'asbab ankhifad darajat altulaab alsueudiiyn fi alriyadiaat fi akhtibar PISA 2018 min wijhat nazar almusharikina [Causes of Saudi students' low PISA 2018 mathematics scores from participants' point of view]. *Journal of Educational Sciences, 32*(3), 589–618.

Al-Hoor, D. (2021). maharat aikhtibar PISA fi kutub aleulum wamustawaa aiktisabiha waealaqatiha biaistikshaf almustaqbal ladaa talabat alsafi altaasie fi mudun aljanub alfilastinii [PISA test skills in science books, the level of acquiring them, and their relationship with future exploration with grade 9 students in southern Palestinian cities] (Master's thesis, Al-Aqsa University, Palestine).

Al-Khadher, N. (2020). tahsin muetaqadat alkafa'at aldhaatiat walmuealimat alriyadiat waeamaliaat althaqafat alriyadiat ladaa almuealimat fi barnamaj altaqyim alduwalii liltulaab [Improving teachers' self-competence beliefs, mathematics knowledge, and mathematics literacy performance in the PISA]. *Studies in Teaching Methods and Curricula, 248*, 18–47.

Al-Towaisi, A. (2022). wujuhat nazar altulaab al'urduniyiyn hawl eawamil tahsin 'adayihim fi barnamaj altaqyim alduwlii liltulaab (PISA) [Jordanian students' perspectives on factors improving their PISA performance]. *International Journal for Research in Educational Sciences, 5*(2), 391–450.

Al-Zahrani, A. (2022). muqtarahat eilajiat litudaniy al'ada' aleilmii liltalabat alsueudiiyn fi PISA 2018 biaistikhdam miqyas dalafi [Remedial suggestions for Saudi students' low science performance in PISA 2018 using a Delphi scale]. *Journal of Islamic University for Educational and Social Sciences, 12*, 255–290.

Atkins, K., Dougan, B., Dromgold-Sermen, S., Potter, H, Sathy, V., & Panter, A. (2020). Looking at myself in the future: How mentoring shapes scientific identity for STEM students from underrepresented groups. *International Journal of STEM Education,* 7, Article 42. https://doi.org/10.1186/s40594-020-00242-3

Cansiz, N., & Cansiz, M. (2019). Evaluating Turkish science curriculum with PISA scientific literacy framework. *Turkish Journal of Education, 8*(3), 217–236. https://doi.org/10.19128/turje.545798

Çikirikçi Demirtasli, N., & Ulutas, S. (2015). A study on detecting of differential item functioning of PISA 2006 science literacy items in Turkish and American samples. *Eurasian Journal of Educational Research, 58*, 41–60. http://dx.doi.org/10.14689/ejer.2015.58.3

Deemer, D., Ogas, P., Barr, C., Bowdon, D., Hall, M. C., Paula, S., Capobianco, B. M., & Lim, S. (2022). Scientific research identity development need not wait until college: Examining the motivational impact of a pre-college authentic research experience. *Research in Science Education, 52*(5), 1481–1496. https://doi.org/10.1007/s11165-021-09994-6

Depren, S. (2018). Prediction of students' science achievement: An application of multivariate adaptive regression splines and regression trees. *Journal of Baltic Science Education, 17*(5), 887–903. doi:10.33225/jbse/18.17.887

Gunaydin, Y., & Basaran, M. (2022). The predictive power of reading comprehension, attitude toward sciences, test technique, and science subject matter knowledge in predicting PISA scientific literacy test total score. *Participatory Educational Research, 9*(6), 206–220. https://doi.org/10.17275/per.22.136.9.6

Hegras, A. (2021). 'athar altadrib ealaa namadhij aikhtibarat PISA fi tahsin 'ada' tulaab alsafi aleashir fi aikhtibar fahm ma hu maktub fi 'iidarat taelim alqasabat fi 'arbadi [The effect of training on samples of PISA tests on improving grade 10 students' performance in testing comprehension of what is written in education administration of Qasaba, Arbad]. *Al-Nagah University Journal for Research, Human Sciences, 35*(2), 199–220.

Hepworth, N., Galvis, M., Gambhir, G., Sizmur, J. (2021). *Using PISA 2018 to inform policy: Learning from the Republic of Ireland*. National Foundation for Educational Research.

Kastberg, D., Murray, G., Ferraro, D., Arieira, C., Roey, S., Mamedova, S., & Liao, Y. (2020). *Technical Report and User Guide for the 2016 Program for International Student Assessment (PISA) young adult follow-up study* (NCES 2021-020). National Center for Education Statistics.

Krippendorf, K. (1980). *Content analysis*. Sage.

Mamedova, S., Stephens, M., Liao, Y., Sennett, J., & Sirma, P. (2021). *2012-2016 Program for International Student Assessment young adult follow-up study (PISA YAFS): How reading and mathematics performance at age 15 relate to literacy and numeracy skills and education, workforce, and life outcomes at age 19* (Research and Development Report NCES 2021-029). National Center for Education Statistics.

Mubarak, M. (2013). al'ada' altafaduliu fi akhtibar alriyadiaat fi PISA 2006 [Differential performance in PISA's 2016 math test]. *Journal of Educational Science, 25*(2), 241–261.

Organization for Economic Cooperation and Development. (2019). *PISA 2018 assessment and analytical framework*. https://read.oecd.org/10.1787/b25efab8-en?format=pdf

Organization for Economic Cooperation and Development. (2020, March). *PISA 2024: Strategic vision and direction for science*. https://www.oecd.org/pisa/publications/PISA-2024-Science-Strategic-Vision-Proposal.pdf

Organization for Economic Cooperation and Development. (2023). *Programme for International Student Assessment (PISA)*. https://www.oecd.org/pisa/

Oseguera, L., Park, H., De Los Rios, M, Aparicio, M., & Johnson, R. (2019). Examining the role of scientific identity in black student retention in a STEM scholar program. *Journal of Negro Education*, *88*(3), 229-248.

Polat, M., Toraman, Ç., & Turhan, N. S. (2022). Reliability analysis of PISA 2018 reading literacy student questionnaire based on item response theory (IRT): Turkey sample: Reliability analysis of PISA 2018 reading literacy. *International Journal of Curriculum and Instruction*, *14*(1), 1004–1028. http://orcid.org/0000-0001-5851-2322

Price, M., Kantrowitz-Gordon, I., & Gordon, E. (2018). Competing discourses of scientific identity among postdoctoral scholars in the biomedical sciences. *CBE - Life Sciences Education*, *7*(2), Article 29. https://doi.org/10.1187/cbe.17-08-0177

Shein, P., Falk, H., & Li, Y. (2019). The role of science identity in science center visits and effects. *Science Education*, *103*(6), 1478–1492. https://doi.org/10.1002/sce.21535

Somakim, S., & Madang, K. (2016). Developing teaching materials PISA-based for mathematics and science of junior high school. *Journal of Education and Practice*, *7*(13), 73–77. https://iiste.org/Journals/index.php/JEP/article/view/30623/31462

Sothayapetch, P., Lavonen, J., & Juuti, K. (2013). A comparative analysis of PISA scientific literacy framework in Finnish and Thai science curricula. *Science Education International, 24*(1), 78–97. http://www.icaseonline.net/sei/march2013/p4.pdf

Turk, O., Ünsal, Y., & Karadag, M. (2018). Examination of assessment and evaluation activities of secondary school 9th and 10th grade physics textbooks in terms of revised Bloom Taxonomy and science literacy competence levels: A sample of Turkey. *European Journal of Education Studies*, *5*(1), 42–55. http://dx.doi.org/10.5281/zenodo.1402228

Young, I. (2018). *The influence of mentoring programs in science and engineering on African American and Latin men's scientific identity* (Publication No. 10981729) [Doctoral dissertation, University of California, Davis]. ProQuest Dissertations and Theses Global.

 Click here to access/download;Figure;Figure one FINAL.docx

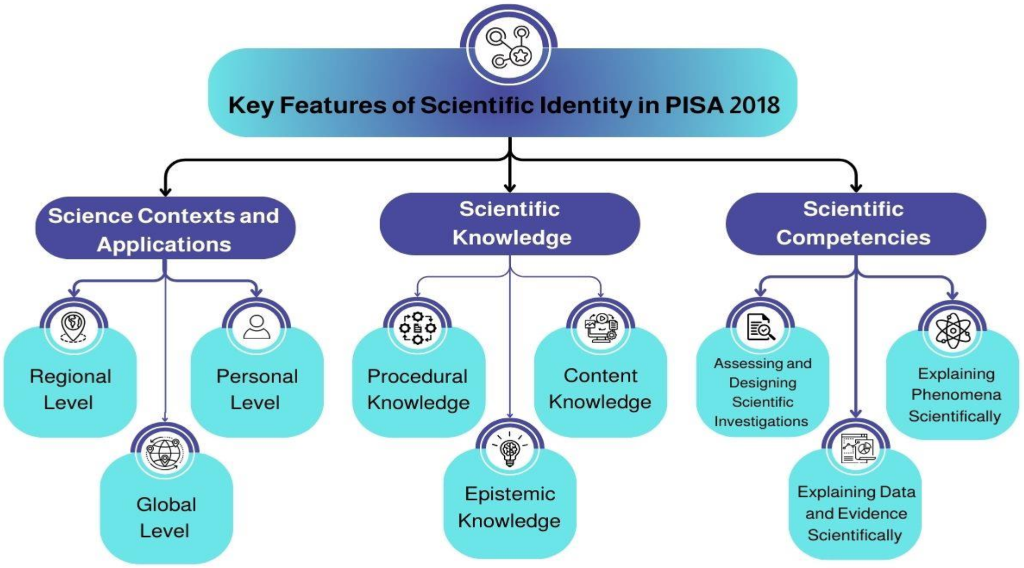

Supplement: Attachment — Submitted filename: PONE-D-25-08148_reviewer Esfand 24th.pdf [file pone.0325542.s003.pdf]
