# Supplementary material for: Saudi secondary school science textbooks’ ability to inculcate a PISA-Informed Scientific Identity
Source: PLoS One. 2025 Oct 6;20(10):e0325542. doi: 10.1371/journal.pone.0325542 (PMC12500081; doi:10.1371/journal.pone.0325542)
Supplement: Attachment — Submitted filename: comments.docx [file pone.0325542.s004.docx]

**Abstract**

The recommendation is not clearly stated even it is good to write in a separate section rather than within the discussion

It does not specify what these revisions might entail.

It mentions definitions is not necessary rather include the scientific concepts are important

Shift after the result of discussing PISA to the textbook analysis is not smoothly shifting or unexpected

**Introduction**

This research is a unique work which focuses on PISA results on Saudi Arabia secondary school indicates its originality.

Although the introduction of this research gives technical foundation and adequate framework of PISA in Saudi Arabia, It does not give detail information about the experiment.

The data is supported by the previous work results but the conclusion is not clearly specified and evidenced.

**Problem Statement, Research Question, and Study Objectives**

Some concepts are not clearly explained. For instance, the relationship of scientific identity on the performance of PISA and the strategies that could help to enhance the achievement of PISA also are not explained. Furthermore, the research gaps are not stated clearly.

It lacks contextual evidences, without supporting the claim that there is lacking research in the area of the research or in Saudi Arabia, it should be supported by references.

It is good that the research question touches the extent of science textbooks but it should include the important issue which is the characteristic of scientific identity with related to the framework of PISA.

**Method**

Although the descriptive analytical method is appropriate, the selecting reason is not justified well and couldn’t answer why grade 9 is selected.

In the identification of PISA dimensions

Scientific dimensions of PISA are not explicitly stated to the importance of the issue of scientific identity.

Recommendation is future work so it had better specify the changes and show how this can be performed.

**Conclusion and implication**

Although the conclusion and implication is clear, it does not show scientific identity can engage students in the contemporary era of technology in the future.

References are well written in APA style
